# Automated Machine Learning (AutoML) for the Diagnosis of Melanoma Skin Lesions From Consumer-Grade Camera Photos

**DOI:** 10.7759/cureus.67559

**Published:** 2024-08-23

**Authors:** Aparna Potluru, Anmol Arora, Ananya Arora, Shaheer Aslam Joiya

**Affiliations:** 1 Dermatology, National Health Service (NHS) Greater Glasgow and Clyde, Edinburgh, GBR; 2 Clinical Medicine, University of Cambridge, Cambridge, GBR; 3 Trauma and Orthopaedics, Yeovil District Hospital, Yeovil, GBR

**Keywords:** skin cancer, dermatology, machine learning in healthcare, artificial intelligence in medicine, melanoma

## Abstract

Background: In recent years, there has been much speculation about the role of artificial intelligence (AI) and machine learning in dermatology. Advances in computer vision have increased the potential for automated diagnosis of images. However, there remains a gap between the technological development of the algorithms and their real-world implementation. This study aims to develop and test an automated machine learning (AutoML) algorithm for the diagnosis of melanoma, with no technical or coding skills required by the operator.

Methods: The Skin Cancer Detection Dataset from the University of Waterloo Vision and Image Processing Lab contains 206 images sourced from the public databases DermIS and DermQuest. The dataset was split into two groups: training data (n=174) and testing data (n=32). A machine learning algorithm was created using ‘Teachable Machine’, trained on the training data, to differentiate between melanoma and non-melanoma skin lesions.

Results: The AutoML algorithm identified 12/14 non-melanoma images and 15/18 melanoma images in the testing dataset. The overall accuracy was 84.4%, with a sensitivity of 83.3% and a specificity of 85.7%.

Conclusions: Existing literature has tested a range of different machine learning algorithms on the same dataset. These have often required expertise in machine learning and the ability to code. The results of this study, using a no-code tool, perform comparably to existing efforts and suggest that there is potential for future clinical AI algorithms to be developed by doctors even without any technical expertise as long as they have access to relevant local data.

## Introduction

The utilisation of artificial intelligence (AI) in healthcare has increased exponentially over the past decade. Its use has been fuelled by the increased availability of digital data, significant enhancements in computing hardware, and innovation in algorithm design [1]. Thus far, computer vision has dominated AI development, with a flurry of research groups finding that AI algorithms were able to analyse images faster and more accurately than humans in some settings [2]. In part due to its reliance on imaging data, dermatology has been at the forefront of computer vision research, with a particular focus on automated diagnosis. Dermatological applications of AI include differentiating between benign and malignant skin lesions, lesion measurement and tracking, automated tissue identification, gene expression profiling, procedure planning, tele-dermatology, and clinical and patient education [3,4].

Despite advances in research in recent years, there is a lack of real-world evidence through randomised controlled trials and prospective studies to support the use of AI in routine clinical practice [5]. This is partly due to unresolved concerns such as generalisability, data requirements, standardisation, and interpretability. It has also been postulated that dermatologists require a basic understanding of AI as a prerequisite to design relevant studies [4]. The assumption that a technical background is required for a dermatologist to develop and interpret an AI model limits the accessibility of AI research. One of the most promising innovations in the field of AI research is the development of automated machine learning (AutoML) systems, which allow users to create AI algorithms without any coding ability required [6]. These often use cloud-based computing to analyse images and produce an algorithm based on data provided by the user. The accuracy metrics produced by AutoML services may compete against algorithms produced by leading AI research groups. If AutoML systems prove successful, there is an opportunity for dermatologists to begin creating their own algorithms using locally collected data without coding expertise. Whilst these AutoML algorithms may not be suitable for use in real-world practice, their use may aid dermatologists in understanding the theory surrounding AI development.

This study uses the University of Waterloo Skin Cancer Detection Dataset, which contains images collected using a consumer-grade camera [7]. We construct and test an AutoML algorithm and compare the results to those obtained by researchers developing their own complex machine learning algorithms. Relevant elements of the STROBE reporting guidelines have been followed. The STROBE guidelines provide a checklist to improve the quality and transparency of reporting in observational studies by ensuring a clear and comprehensive presentation of essential study elements, such as design, participant selection, data collection, and analysis.

## Materials and methods

University of Waterloo dataset

The Skin Cancer Detection Dataset from the University of Waterloo Vision and Image Processing Lab contains 206 images sourced from the public databases DermIS and DermQuest. Processed images with lesion borders labelled by manual segmentation were available but not used. The dataset comprises 87 non-melanoma images and 119 melanoma images. The dataset was chosen because images were taken using a consumer-grade camera, facilitating the opportunity for researchers to replicate findings in the future with novel data using non-specialist equipment.

Inclusion and exclusion criteria

Only raw images from the dataset were included in the study. Processed images with lesion borders labelled by manual segmentation were available but were excluded to ensure the machine learning model was trained on unprocessed data, reflecting real-world scenarios where such pre-processing may not be present. Demographic data on the patients is not available.

Study parameters

Dataset Splitting

The dataset was divided into two groups: 174 images were used for training, and 32 images were reserved for testing. This split followed an approximately 85:15% ratio to provide a robust training set whilst reserving a portion of the data for unbiased performance evaluation.

AutoML algorithm

Algorithm Selection

The Teachable Machine platform, a no-code online service, was employed to create the machine learning algorithm [8,9]. This platform offers users the flexibility to customise training parameters, including the number of epochs, batch size, and learning rate.

Model training

For this study, the training parameters were set to 60 epochs, a batch size of 16, and a learning rate of 0.0005. The model was trained using the 174 images in the training dataset.

The dataset was split into two groups: training data (n=174) and testing data (n=32) in an approximately 85:15% ratio. The model was trained on the testing data, and then its performance was tested on the previously unseen testing data.

## Results

Of the 32 images allocated to the testing dataset, the machine learning algorithm correctly identified 27 (accuracy = 0.84). Of the 14 non-melanoma images for testing, the algorithm correctly identified 12 (specificity = 0.86). Of the 18 melanoma images, the algorithm correctly identified 15 (sensitivity = 0.83) (Table 1). These results are illustrated as a confusion matrix in Table 2.

**Table 1:** Comparisons of the results of this paper against previous attempts using the same dataset AutoML: automated machine learning

Author	Model	Description	Accuracy	Sensitivity	Specificity
Amelard et al. (2015) [10]	S_T_ feature set (F_1_ function)	Uses a set of 62 features extracted from the images	83.59	91.01	73.45
Amelard et al. (2012) [11]	F_T_ feature set	Uses a set of 51 features extracted from the images	87.38	90.76	82.76
Amelard et al. (2013) [12]	F_T_ feature set	Uses a set of 59 features extracted from the images	81.26	84.04	79.91
Haider et al. (2014) [13]	S_LHP_ feature set	Uses a hybrid set of low-level features, high-level features, and physiological features	83.05	87.73	76.34
Arora et al. (this paper)	AutoML (via Teachable Machine)	No-code AutoML model	84.4	83.3	85.7

**Table 2:** Confusion matrix illustrating the results of the AutoML algorithm for melanoma detection AutoML: automated machine learning

Actual lesion classification	Non-melanoma	12	2
	Melanoma	3	15
		Non-melanoma	Melanoma
		Model prediction	

These findings suggest that the algorithm performs reliably in distinguishing between melanoma and non-melanoma images, achieving a balance between sensitivity and specificity. The high specificity indicates the algorithm's effectiveness in correctly identifying non-melanoma cases, minimising false positives. Conversely, the sensitivity result reflects the algorithm’s ability to detect true melanoma cases, although some false negatives were present. The overall accuracy of 0.84 underscores the algorithm’s robust performance in this initial study, especially given the complexities inherent in dermatological image classification.

## Discussion

To the best of our knowledge, this preliminary study represents the first reported use of AutoML on the University of Waterloo Skin Cancer Detection Dataset. Respectable levels of accuracy were obtained using a no-code AI development tool. This democratisation of AI research is crucial, as it empowers non-technical professionals, such as dermatologists, to develop localised AI algorithms tailored to their specific clinical environments. However, whilst these results are promising, they also underscore the complexity and challenges that still need to be addressed in this field. Furthermore, the levels of accuracy were based on image analysis alone. Future efforts may aim to combine elements of the clinical history as well, including the rate of change of the lesion, the presence of symptoms, and the surrounding clinical context. Such multiparametric analysis may yield higher levels of accuracy and more closely resemble the diagnostic reasoning process of a human dermatologist [1].

The use of AutoML, particularly no-code platforms, represents a paradigm shift in AI research and application. Traditionally, developing machine learning models required extensive technical expertise and resources, often limiting these tools to larger institutions with dedicated data science teams. The ability of non-technical professionals to develop AI models is a crucial step in bridging the gap between advanced technology and clinical practice. This approach aligns with the broader trend of democratising AI, making it accessible to a wider audience, and fostering innovation in diverse fields, including dermatology.

Whilst the study primarily focused on image-based analysis, the suggestion to integrate additional clinical parameters is particularly compelling. In clinical practice, dermatologists do not rely solely on visual information; they consider a wide range of factors, including the lesion's history, rate of change, associated symptoms, and broader clinical context. Incorporating these elements into machine learning models could significantly enhance diagnostic accuracy, making AI tools more reflective of the holistic diagnostic processes used by human experts. Recent research has highlighted the potential of such multiparametric models, which integrate clinical and dermoscopic data, to improve diagnostic performance. For instance, studies have shown that combining clinical history with imaging data can help distinguish between benign and malignant lesions with greater precision.

The results obtained from the no-code AutoML solution are comparable to results from much more sophisticated methods of machine learning development. Such comparisons are noted in Table 2 [10-13]. This finding is particularly significant as it challenges the notion that advanced technical methods are always necessary for achieving high levels of accuracy. However, it is important to note that comparisons between different studies are complicated by the variability in data allocation for training and testing, as well as differences in dataset composition. As there is no fixed allocation of data for training and testing and the proportional allocation to the two datasets is variable between research groups, these are only approximate comparisons. Therefore, whilst these findings are encouraging, further research is needed to standardise evaluation methods and ensure that comparisons across studies are meaningful.

Although the findings are promising, there are a number of limitations. Firstly, the demographic information of the patients represented in the dataset is not available. However, from a basic inspection of the data, there is little diversity in skin tone, and the images are generally of high quality. This is unlikely to be generalisable to real-world data, although the images were obtained using only a consumer-grade camera, which is likely to make the findings more accessible [14]. A common problem with machine learning algorithms is their black-box nature, whereby it is difficult to inspect the reasoning and understand how the algorithms reach their conclusions. Although the inputs and outputs of the algorithm are observable, the algorithmic reasoning through which the predictions are formed is hidden. Thus, it is not possible to inspect the rationale for a suggested diagnosis in the way that may be possible for a human diagnosis. This limits the amount of trust that can be placed in the algorithm's performance. For example, there is no way to inspect whether the algorithm is systematically failing at diagnosing specific subtypes of melanoma, in this case, because the dataset is not labelled accordingly. Atypical melanomas or those that require tactile information may be undetectable by a machine learning algorithm trained on such imaging data. Emerging research in explainable AI is attempting to address this issue by developing methods that make AI decision-making processes more transparent and interpretable.

This study was also limited by the size of the dataset, as a larger training dataset would have likely improved model performance. Larger datasets typically allow for more robust model training and validation, leading to improved performance. In addition to size, the diversity of the dataset is crucial. This study sought to simply identify the presence of a melanotic skin lesion but was not able to diagnose the remaining lesions beyond stating that they were non-melanotic. This was due to the lack of labelling of such lesions in the dataset. A more diverse dataset, including a broader range of skin tones and lesion types, would likely enhance the model's generalizability. This is particularly important given the global burden of skin cancer, which affects individuals of all racial and ethnic backgrounds. Future studies should prioritise the collection of diverse, high-quality data to ensure that AI models can serve all populations effectively.

The study was primarily focused on identifying melanotic lesions, which are a significant concern in dermatology due to their potential for malignancy. However, the inability to diagnose other types of lesions highlights a critical gap in the current approach. To fully leverage AI in dermatology, future research should aim to develop models capable of diagnosing a broader spectrum of skin conditions. Recent advancements in multi-class classification algorithms and the integration of dermoscopic data with clinical images have shown promise in this area [1]. Expanding the scope of AI models to include various skin conditions will make these tools more useful in clinical practice, where dermatologists encounter a wide range of skin pathologies.

## Conclusions

Overall, this study has demonstrated the potential for no-code AutoML tools to reach respectable levels of accuracy for the diagnosis of melanoma from standard skin images. With no-code tools, AI research is becoming increasingly accessible to dermatologists without technical expertise. Due to the limitations of machine learning, it would be advisable, however, to seek advice from experts in order to minimise the risk of adverse outcomes, including algorithmic bias. Future studies may seek to use larger datasets or augment existing datasets through the production of synthetic data. Additional research is required to validate these findings, as there is a notable lack of randomised controlled trials or prospective studies assessing the use of AI in dermatological practice. The ultimate goal is to create AI systems that not only replicate but also augment the diagnostic capabilities of human dermatologists, leading to improved patient outcomes across diverse populations.
